# A plasma circulating miRNAs profile predicts type 2 diabetes mellitus and prediabetes: from the CORDIOPREV study

**DOI:** 10.1038/s12276-018-0194-y

**Published:** 2018-12-26

**Authors:** Rosa Jiménez-Lucena, Antonio Camargo, Juan Francisco Alcalá-Diaz, Cristina Romero-Baldonado, Raúl Miguel Luque, Ben van Ommen, Javier Delgado-Lista, Jose María Ordovás, Pablo Pérez-Martínez, Oriol Alberto Rangel-Zúñiga, Jose López-Miranda

**Affiliations:** 1 Lipids and Atherosclerosis Unit, Reina Sofıa University Hospital, Córdoba, Spain; 2 IMIBIC/Reina Sofía University Hospital, University of Córdoba and CIBER Fisiopatología de la Obesidad y la Nutrición (CIBEROBN), Instituto de Salud Carlos III, Madrid, Spain; 3 Biochemical Laboratory, Reina Sofia University Hospital, Córdoba, Spain; 4 Department of Cell Biology, Physiology, and Immunology, Agrifood Campus of International Excellence (ceiA3), University of Córdoba, Cordoba, Spain; 5 Netherlands Institute for Applied Science (TNO), Research Group Microbiology and Systems Biology, Zeist, The Netherlands; 6 Nutrition and Genomics Laboratory, J.M, US Department of Agriculture Human Nutrition Research Center on Aging, Tufts University, Boston, MA USA; 7 Centro Nacional de Investigaciones Cardiovasculares, Madrid, Spain; 8 IMDEA Food Institute, CEI UAM+CSIC, Madrid, Spain

## Abstract

We aimed to explore whether changes in circulating levels of miRNAs according to type 2 diabetes mellitus (T2DM) or prediabetes status could be used as biomarkers to evaluate the risk of developing the disease. The study included 462 patients without T2DM at baseline from the CORDIOPREV trial. After a median follow-up of 60 months, 107 of the subjects developed T2DM, 30 developed prediabetes, 223 maintained prediabetes and 78 remained disease-free. Plasma levels of four miRNAs related to insulin signaling and beta-cell function were measured by RT-PCR. We analyzed the relationship between miRNAs levels and insulin signaling and release indexes at baseline and after the follow-up period. The risk of developing disease based on tertiles (T1-T2-T3) of baseline miRNAs levels was evaluated by COX analysis. Thus, we observed higher *miR-150* and *miR-30a-5p* and lower *miR-15a* and *miR-375* baseline levels in subjects with T2DM than in disease-free subjects. Patients with high *miR-150* and *miR-30a-5p* baseline levels had lower disposition index (*p* = 0.047 and *p* = 0.007, respectively). The higher risk of disease was associated with high levels (T3) of *miR-150* and *miR-30a-5p* (*HR*_*T3-T1*_ = 4.218 and *HR*_*T3-T1*_ = 2.527, respectively) and low levels (T1) of *miR-15a* and *miR-375* (*HR*_*T1-T3*_ = 3.269 and *HR*_*T1-T3*_ = 1.604, respectively). In conclusion, our study showed that deregulated plasma levels of *miR-150*, *miR-30a-5p*, *miR-15a*, and *miR-375* were observed years before the onset of T2DM and pre-DM and could be used to evaluate the risk of developing the disease, which may improve prediction and prevention among individuals at high risk for T2DM.

## Metabolic disease: a red flag for diabetes risk

Tiny RNA molecules circulating in the blood could give early warning of type 2 diabetes risk. MicroRNAs help regulate the expression of other genes, and recent research has linked irregularities in these molecules to many different diseases. Researchers led by José López Miranda of the University of Córdoba in Spain monitored a cohort of 462 patients for several years to assess how plasma levels of certain microRNAs are deregulated before the onset and progression of diabetes. They observed a striking ‘signature’ of altered expression in four microRNAs for patients who developed diabetes over the course of the study. Intriguingly, patients with markedly elevated blood sugar—state known as prediabetes—exhibited a similar signature, but with more modest alteration in the gene expression levels, indicating that these microRNAs could help clinicians track and prevent disease onset.

## Introduction

Type 2 diabetes mellitus (T2DM) is a major public health problem, and its prevalence has been increasing over the past few decades with no signs of receding in the near future. In addition to personal affliction, this disease imposes a heavy economic burden on the global health-care system and overall economy^1^.

In prediabetic conditions, beta cells compensate the insulin-resistant state from target tissues by increasing both the mass of pancreatic islets and the release of insulin in response to dietary glucose and fatty acids^2^. Chronic stress conditions may induce the activation of adaptation processes that exceed the normal phenotypic flexibility, leading to progressive inflexibility^3^ and beta-cell dysfunction, followed by elevated fasting glucose (IFG) and/or impaired glucose tolerance (IGT)^4,5^. Thus, the degree of hyperglycemia reflects the severity of the metabolic process, suggesting that it is an important marker to determine the developmental stage of the disease^6,7^.

However, T2DM frequently goes undiagnosed for many years because hyperglycemia develops gradually and, in its earlier stages, is not severe enough for the patient to note any of the classic symptoms of diabetes^8^. For this reason, the tools used currently to screen and diagnose T2DM and to detect individuals with prediabetes (Pre-DM) do not adequately predict the onset of the disease and monitor its progression^9^. Therefore, more informative biomarkers are needed to identify beta-cell injury and to assess the risk of disease, monitor responses to treatment, personalize therapy and improve patient quality of life^10^.

Currently, miRNAs are recognized as important regulators of gene expression and central players in the control of several biological and pathological processes, including T2DM^11^. To this end, in vitro models have shown that *miR-30a-5p*^12^ and *miR-144* play a role in beta-cell dysfunction^13^. Moreover, the plasma concentrations of individual^14^ and multiple circulating miRNAs have been identified as being significantly different between T2DM patients and prediabetic subjects^15,16^. In addition, alterations in plasma miRNAs levels have been observed in other metabolic diseases, such as metabolic syndrome^15,17^. However, those experimental designs were not able to identify whether the differences in miRNA levels found were the cause or a consequence of the development of metabolic disease. To the best of our knowledge, only two studies have observed alterations in plasma miRNAs levels before T2DM development^18,19^.

This imparts a need to conduct long-term follow-up studies in larger populations with incident cases of T2DM to identify the role of miRNA during the development of T2DM and to use that information to assist in the development of predictive biomarkers of T2DM. Therefore, in this study, we aimed to study whether plasma circulating levels of miRNAs according to T2DM or pre-DM status could be used as biomarkers to evaluate the risk of developing the disease.

## Materials and methods

### Study subjects

This work was conducted within the framework of the CORDIOPREV study, whose rationale, methods and baseline characteristics have been reported by Delgado-Lista et al.^20^. Briefly, the CORDIOPREV study is an ongoing prospective, randomized, single-blind, controlled dietary intervention trial in 1002 coronary health disease (CHD) patients with a high risk of cardiovascular disease who had their last coronary event more than 6 months before enrollment. In addition to conventional treatment for CHD, the subjects were randomized into two different dietary models (Mediterranean and low-fat diets). The intervention phase is still in progress and will have a median follow-up of seven years. Patients were recruited from November 2009 to February 2012, mostly at the Reina Sofia University Hospital (Córdoba, Spain), but patients from other hospitals from the Córdoba and Jaen provinces were also admitted. In summary, the patients were eligible if they were between 20 and 75 years of age, had established CHD without any clinical events in the last 6 months, were thought to follow a long-term dietary intervention and had no severe diseases or a life expectancy of five years. Details of the trial design were provided in Clinicaltrials.gov (NTC00924937). Written consent was obtained from all subjects prior to recruitment and initiation of the study protocol, and all the amendments were approved by the Ethics Committee of Hospital Reina Sofia, all of which follow the Helsinki Declaration and good clinical practices.

All patients from the CORDIOPREV-DIAB without T2DM at baseline according to the American Diabetes Association (ADA) diagnostic criteria^21^ (*N* = 462) were included in this study^22^. T2DM and pre-DM were diagnosed according to the ADA diagnostic criteria^21^ and were evaluated by glucose tolerance tests performed each year during the five years of follow-up. Prediabetic status was defined as having one or more of the following criteria present in a participant: fasting plasma glucose (FPG) concentration ≥100 and <126 mg/dL, impaired fasting glucose (IFG); FPG ≥ 140 and <200 mg/dL 2 h after an oral glucose test (OGTT), impaired glucose tolerance (IGT)^23^ and glycosylated hemoglobin (HbA1c) ≥5.7 and <6.4%^24^.

In a median follow-up of 60 months, 78 subjects did not develop T2DM or pre-DM (non-T2DM); 239 subjects were pre-DM at baseline, of which 223 subjects maintained pre-DM status during the follow-up period (pre-DM); 30 subjects were not pre-DM at baseline but developed pre-DM in the follow-up period (incident pre-DM). Finally, 107 of the participating subjects developed T2DM (incident-T2DM) in a median follow-up of 60 months. Moreover, 24 patients were not included in this study due to declining participation, death and withdrawing for other reasons. The baseline characteristics of the subjects in the study are shown in Table 1.

**Table 1:** Baseline characteristics of different groups of patients included in the study

	Non-T2DM	Pre-DM	Incident Pre-DM	Incident-T2DM	*p*-value
*N*	78	223	30	107	
Age (years)	**53** **±** **1**	**59** **±** **1**^a^	**57** **±** **2**	**59** **±** **1**^c^	<0.001*
BMI (kg/m^2^)	**28.9** **±** **0.4**	**30.3** **±** **0.3**	**29.3** **±** **1.0**	**31.4** **±** **0.5**^c^	0.001*
Waist circumference (cm)	**99.1** **±** **1.1**	**102.8** **±** **0.7**^a^	**99.1** **±** **2.3**	**105.3** **±** **1.1**^c^	<0.001*
Total cholesterol (mg/dL)	**162** **±** **3**	**160** **±** **2**	**147** **±** **6**	**165** **±** **3**	0.042*
c-HDL (mg/dL)	46.2 **±** 1.2	44.0 **±** 0.7	43.2 **±** 2.0	43.5 **±** 1.0	0.236
TG (mg/dL)	**125** **±** **9**	**120** **±** **4**	**94.5** **±** **8.4**	**133** **±** **7**	0.011*
c-LDL (mg/dL)	91.5 **±** 2.8	91.7 **±** 1.7	82.8 **±** 3.6	93.4 **±** 2.7	0.301
hs-CRP (mg/L)	2.37 **±** 0.44	2.57 **±** 0.24	1.98 **±** 0.36	2.88 **±** 0.29	0.066
HbA1c (%)	**5.66** **±** **0.04**	**5.97** **±** **0.02**^a^	**5.43** **±** **0.04**^b^	**6.03** **±** **0.03**^c^	<0.001*
Glucose (mg/dL)	**88.8** **±** **1.1**	**94.0** **±** **0.7**^a^	**88.1** **±** **1.2**	**96.2** **±** **1.0**^c^	<0.001*
Insulin (mU/L)	**8.15** **±** **0.69**	**8.57** **±** **0.39**	**7.83** **±** **0.88**	**10.51** **±** **0.66**	0.017*
Glucose 2 h after OGTT (mg/dL)	**110** **±** **4**	**125** **±** **2**^a^	**113** **±** **4**	**135** **±** **3**^c^	<0.001*
ISI	**5.02** **±** **0.30**	**4.02** **±** **0.18**^a^	**4.36** **±** **0.45**	**3.27** **±** **0.19**^c^	<0.001*
HOMA-IR	**2.22** **±** **0.17**	**2.71** **±** **0.12**	**2.26** **±** **0.19**	**3.23** **±** **0.22**^**c**^	0.001*
HIRI	**912** **±** **71**	**1102** **±** **46**	**932** **±** **77**	**1313** **±** **90**^**c**^	0.002*
IGI	**1.04** **±** **0.09**	**1.16** **±** **0.08**	**1.42** **±** **0.19**	**0.88** **±** **0.07**	0.038*
DI	**1.14** **±** **0.06**	**0.93** **±** **0.04**^a^	**1.27** **±** **0.11**	**0.77** **±** **0.04**^**c**^	<0.001*
MISI (×10^2^)	2.48 **±** 0.28	2.01 **±** 0.14	1.54 **±** 0.19	1.89 **±** 0.16	0.125

Subjects were classified according to pre-DM and T2DM development status after a follow-up median of 60 months. Values are expressed as the mean ± standard error. Variables were calculated by ONE-WAY ANOVA using SPSS (now PASW Statistics for Windows (version 21.0)) (IBM. Chicago, Illinois). Subjects did not develop T2DM or pre-DM (non-T2DM); subjects maintained pre-DM during the follow-up period (pre-DM); subjects not pre-DM at baseline but developed pre-DM in the follow-up period (Incident pre-DM); and subjects developed T2DM (Incident-T2DM).

*BMI* Body mass index, *c-HDL* High density lipoprotein, *c- LDL* Low density lipoprotein, *TG* Triglycerides, *hs-CRP* High sensitivity C-reactive protein, *HbA1c* Glycosylated hemoglobin, *HIRI* Hepatic insulin resistance index, *MISI* muscle insulin sensitivity index, *ISI* insulin sensitivity index, *IGI* insulinogenic index, *DI* disposition index, *HOMA-IR* homeostasis model assessment- insulin resistance,

*Statistically significant differences (*p* < 0.05) are in bold

^a^p < 0.05 non-T2DM vs. pre-DM subjects in the Post Hoc analysis

^b^p < 0.05 non-T2DM vs. incident pre-DM subjects in the Post Hoc analysis

^c^p < 0.05 non-T2DM vs. incident-T2DM subjects in the Post Hoc analysis

### Biochemical measurements of metabolic parameters

Venous blood from the participants was collected in tubes containing EDTA after a 12-h overnight fast. Lipid variables were assessed with the modular auto analyzer DDPPII Hitachi (Roche, Basel, Switzerland) using specific reagents (Boehringer-Mannheim, Mannheim, Germany). Measurements of total cholesterol (TC) and triglycerides (TG) levels were made by colorimetric enzymatic methods^25,26^; high-density lipoprotein-cholesterol (HDL-c) levels were measured by colorimetric assay^27^; and low-density lipoprotein (LDL-C) concentrations were calculated by the Friedewald equation, using the following formula: LDL-C = CT - (HDL-C + TG/5). Glucose measurements were performed using the hexokinase method. hs-C-reactive protein (hs-CRP) concentration was determined by high-sensitivity ELISA (BioCheck, Inc., Foster City, CA, USA). Plasma insulin concentrations were measured by microparticle enzyme immunoassay (Abbott Diagnostics, Matsudo-shi, Japan). Non-esterified fatty acid concentrations were measured by enzymatic colorimetric assay (Roche Diagnostics, Penzberg, Germany). ApoA-1 and ApoB concentrations were determined by immunoturbidimetry.

### Insulin signaling and release indexes

Before starting the test, the patients abstained from food and medications for 12 h and were asked to refrain from smoking during the fasting period and from alcohol intake during the preceding seven days. They were also requested to avoid strenuous physical activity the day before the test was administered. Oral glucose tolerance test (OGTT): At 8:00 a.m., the patients were admitted to the laboratory, and an OGTT (75 g dextrose monohydrate in 250 mL water, NUTER. TEC GLUCOSA 50) was performed with 0, 30, 60, and 120 min sampling to establish plasma glucose and insulin levels^28^.

The insulin sensitivity index (ISI) was calculated in the OGTT using the following formula: ISI = 10.000 ÷ √([fasting plasma glucose × fasting plasma insulin]×[mean glucose in OGTT × mean insulin in OGTT])^28^. HOMA-IR was calculated as previously described by Song, Y., et al.^29^. Insulin secretion was measured using the insulinogenic index (IGI): IGI = [30 min insulin−fasting insulin (pmol/L)]/[30 min glucose−fasting glucose (mmol/L)^30^. The disposition index (DI) was estimated as follows: DI = ISI × [AUC30 min insulin/AUC30 min glucose], where AUC30 min is the area under the curve between the baseline and 30 min of the OGTT for insulin (pmol/L) and glucose (mmol/L) measurements, respectively, calculated by the trapezoidal method^31^. The hepatic insulin resistance index (HIRI) and the muscle insulin sensitivity index (MISI) were used to evaluate tissue-specific IR as described in previous work by our group^22^, following the methods described by Matsuda and DeFronzo for HIRI and Abdul-Ghani et al. for MISI^28,32^.

### Isolation of circulating miRNAs

Circulating miRNAs were isolated from plasma samples, which were obtained using an EDTA-containing blood tube. In this way, venous blood from the participants was obtained by venipuncture and kept on ice until centrifugation. Immediately, to separate the plasma from the erythrocytes and the buffy coat fractions, EDTA-containing blood tubes were centrifuged at 2.000×*g* for 10 min at 4 °C. The superior phase corresponding to the plasma was carefully separated using a pasteur pipette, and the sample was stored at −80 °C until use. The plasma samples were then thawed on ice for RNA isolation.

Total RNA from plasma was isolated using an miRNeasy Mini Kit (Qiagen, Hilden, Germany). In short, 200 µL of EDTA-plasma was mixed with 1 mL of Qiazol, incubated for 5 min at room temperature and then mixed with 200 µL of chloroform. We added 2 μg of MS2 RNA carrier (Roche. Mannheim, Germany) before the chloroform protocol step. The organic and aqueous phases were separated by centrifugation at 12,000×*g* for 15 min at 4 °C. The aqueous phase was collected, and RNA was precipitated by adding 100% ethanol. The mixture was applied to an miRNeasy Mini spin column and centrifuged at 8000×*g* for 2 min. Next, 700 µL of RWT buffer was added to an RNeasy MinElute spin column at 8000×*g* for 2 min. The contents in the column were washed with 500 µL of RPE buffer and 500 µL of 80% ethanol. RNA was eluted in 14 μL of RNase-free water. RNA purity and concentration were evaluated by spectrophotometry using a NanoDrop ND-2000 (ThermoFisher, Waltham, MA).

### Retrotranscription and preamplification of miRNAs

The study of miRNAs expression was carried out on four miRNAs (*miR-150; miR-30a-5p; miR-15a and miR-375)* selected on the basis of previous evidence of their participation in the biomechanisms involved in T2DM pathogenesis–in particular, beta cell function, insulin secretion and insulin sensitivity^12,33–37^. The retrotranscription of RNA was carried out using a TaqMan MicroRNA Reverse Transcription Kit (Life Technologies, Carlsbad, CA, USA). The RT mix contained 2 μL of RNA and 3 µL of RT custom primer pool in a final volume of 7.5 μL. The RT primer pool was customized by selecting specific primers for our set of target miRNAs in the database (https://www.thermofisher.com/es/en/home/life-science/pcr/real-time-pcr/real-time-pcr-assays/mirna-ncrna-taqman-assays.html). The plates were incubated in an iQ5 thermocycler (Bio-Rad Laboratories, Inc., Hercules, CA, USA) at 16 °C for 30 min, followed by 42 °C for 30 min and, finally, 85 °C for 5 min. In this step, the cDNA could be stored at −20 °C for one week. We then prepared a mixture containing 10 μL of a PreAmp custom primer pool specific to our set of target miRNAs, 7.5 µL of RT mix and 20 μL of TaqMan PreAmp Master Mix (Life Technologies, Carlsbad, CA, USA) to make a final volume of 40 µL. The mixture was then incubated in a iQ5 Thermocycler under the following conditions: denaturation at 95 °C for 10 min; 55 °C for 2 min, 72 °C for 2 min; 20 cycles of amplification (15 s at 95 °C, 4 min at 60 °C); and finally 99.9 °C for 10 min. The preamplified products were then diluted with RNase-free water at a ratio of 1:40 and used for real-time RT-PCR reactions.

### Measuring levels of circulating miRNAs using real-time PCR

The circulating levels of miRNAs were measured using the OpenArray® platform (Life Technologies, Carlsbad, CA, USA) following the manufacturer’s instructions. As a normalization method, we first selected the miRNAs that showed the least variability in their CT values in all samples. For this, we used the NormFinder Bioinformatic tool (MOMA-Department of Molecular Medicine, Aarhus University Hospital, Denmark)^38^ (software extensively used in expression studies^39,40^. The application showed that the most stable miRNAs were *miR-143* and *miR-144*. Second, we used the BestKeeper method to calculate the geometric mean of the pair-wise Ct values (Ct values of *miR-143* and *miR-144*)^41^. The relative expression data were analyzed by OpenArray® Real-Time qPCR Analysis Software and with the relative quantification powered by Thermo Fisher Cloud (Life Technologies, Carlsbad, CA, USA). miRNAs levels are expressed in arbitrary units (AU), calculated with the Ct of each target miRNA with reference to the BestKeeper value.

### Relationship of insulin signaling and release indexes with circulating miRNA levels

To analyze the relationship between baseline plasma miRNA levels and both insulin signaling and release indexes, the subjects were categorized by the median of their plasma miRNA levels. Thus, for each miRNA studied, two groups were created: one low-level group and one high-level group. The evolution of signaling and release indexes was evaluated between baseline and a median follow-up of 60 months, according to the levels of miRNAs expression (low or high).

### Statistical analysis

The quantitative data were evaluated using the Kolmogorov–Smirnov test to determine whether they followed a normal distribution. The parameters were taken as normally distributed if *p* > 0.05. A comparative analysis of baseline plasma miRNAs levels between the pre-DM or T2DM groups was carried out using ONE-WAY ANOVA. Statistical significance for non-normal distribution variables was assessed by non-parametric tests using the Mann–Whitney *U* test. The effect of baseline levels of miRNAs on insulin resistance, insulin secretion and DI after a median follow-up of 60 months was assessed using the Wilcoxon test. Non-T2DM, pre-DM and incident pre-DM subjects were grouped, and the probability of developing T2DM (incident-T2DM) was evaluated by Cox regression analysis to test the potential predictive value of the miRNAs studied. The subjects were categorized into tertiles of plasma levels for each miRNA analyzed. Thus, low (T1), intermediate (T2) and high (T3) plasma levels were the three groups defined. The hazard ratio (*HR*) in the Cox regression analysis of each miRNA studied was analyzed by comparing T1 vs. T2 and T1 vs. T3. *P* < 0.05 was considered to be significant. All analyses were adjusted for age, diet, gender, body mass index, glycosylated hemoglobin, HDL-C, triglycerides and waist circumference. Receiver operating characteristic curve (ROC) analysis was performed after including the study variables in a logistic binary regression analysis and with the statistical residuals (probabilities). The statistical analysis was carried out using SPSS (now PASW Statistics for Windows (version 21.0) (IBM. Chicago, Illinois, USA).

## Results

### Baseline characteristics of patients

In a median follow-up of 60 months, 78 subjects did not develop T2DM or pre-DM (non-T2DM); 239 subjects were pre-DM at baseline, of which 223 subjects maintained pre-DM during the follow-up period (pre-DM); 30 subjects were not pre-DM at baseline but developed pre-DM in the follow-up period (Incident pre-DM). Finally, 107 of the participating subjects developed T2DM (Incident-T2DM) in a median follow-up of 60 months. BMI, waist circumference, total cholesterol, TG, HbA1c, fasting glucose, insulin, glucose after 2 h of OGTT, HOMA-IR and HIRI were all higher in incident-T2DM than in pre-DM, incident pre-DM and non-T2DM subjects (all *p* < 0.05). In contrast, ISI, IGI, and DI were lower in incident-T2DM subjects than in pre-DM subjects and non-T2DM subjects after a median follow-up of 60 months (Table 1).

### Baseline levels of circulating miRNAs in the study

**Fig. 1:**
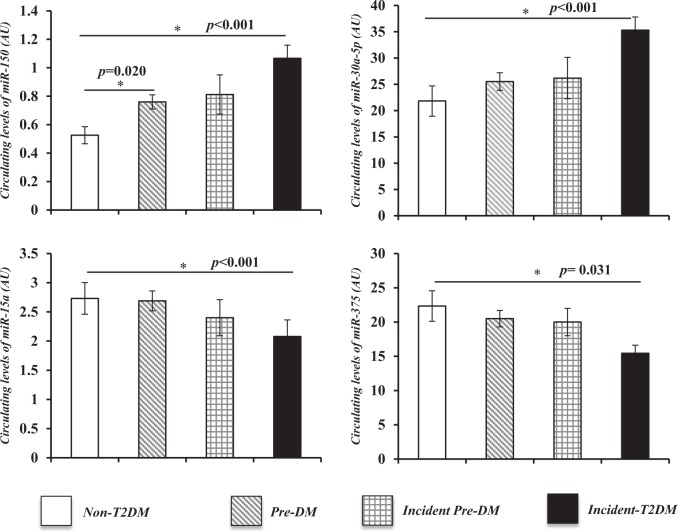
Baseline levels of miRNAs studied according to pre-DM or T2DM status at a median follow-up of 60 months. Values are expressed as the mean ± standard error. Variables were calculated using ONE-WAY ANOVA through SPSS (now PASW Statistics for Windows (version 21.0) (IBM. Chicago, Illinois)). Subjects did not develop T2DM or pre-DM (non-T2DM); subjects maintained pre-DM during the follow-up period (pre-DM); subjects not pre-DM at baseline but developed pre-DM in the follow-up period (Incident pre-DM); and subjects developed T2DM (Incident-T2DM). Significance was assessed by non-parametric tests using the Mann-Whitney *U* test. * *p* < 0.05

We observed that baseline plasma levels of *miR-150* and *miR-30a-5p* were higher and that *miR-15a* and *miR-375* were lower in incident-T2DM subjects than in non-T2DM subjects, with intermediate values in the pre-DM and incident pre-DM patients (*p* < 0.001; *p* < 0.001; *p* = 0.001; and *p* = 0.044, respectively). We also observed that the plasma levels of *miR-150* were higher in the pre-DM subjects than in non-T2DM subjects (*p* = 0.020) (Fig. 1).

### Relationship between miRNAs levels and insulin signaling and release indexes

**Fig. 2:**
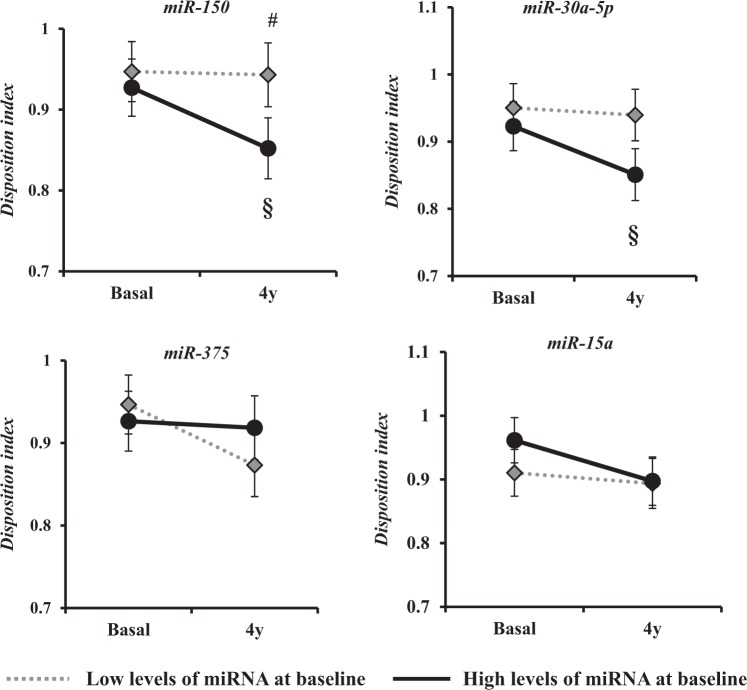
Disposition index changes according to median levels of miRNAs. Values are expressed as the mean ± standard error. Variables were calculated by Wilcoxon test using SPSS (now PASW Statistics for Windows (version 21.0) (IBM. Chicago, Illinois)). The dotted gray line shows subjects with low levels of miRNA at baseline, and the continuous black line shows subjects with high levels of miRNA at baseline. §*p* < 0.05 baseline vs. 4 y. #*p* < 0.05 Low expression levels vs. high expression levels groups

We studied the changes according to the baseline miRNAs levels in the insulin signaling and release indexes (DI, ISI, HIRI and MISI) after 4 years of follow-up. The subjects were categorized according to the median basal plasma levels for each of the four miRNAs studied. We observed that DI decreased after 4 years in subjects with high plasma levels of *miR-150* and *miR-30a-5p* (*p* = 0.047 and *p* = 0.007, respectively) compared with subjects with low plasma levels of these miRNAs. In contrast, no significant differences were observed in *miR-375* and *miR-15a* (Fig. 2). The DI was lower in patients with high plasma levels of *miR-150* compared with that in patients with low plasma levels for this miRNA at 4 years of follow-up (*p* = 0.029) (Fig. 2).

Moreover, the ISI and MISI indexes decreased after the follow-up period in patients with high baseline plasma levels of *miR-150* (*p* < 0.001 and *p* = 0.020, respectively) when compared with the low plasma levels group (Table 2). Likewise, patients with high baseline plasma levels of *miR-375*, compared to the group with low levels of this miRNA, showed a decrease in the HIRI after the follow-up period (*p* < 0.001) (Table 2).

**Table 2:** T2DM-related indexes between baseline and 4 years of follow-up divided by the median of baseline circulating miRNAs

		miR-150 levels		miR-30a-5p levels		miR-15a levels		miR-375 levels	
		low	high	low	high	Low	high	low	high
DI	**0** **y**	**0.95** **±** **0.03**	**0.93** **±** **0.03**	**0.95** **±** **0.03**	**0.92** **±** **0.03**	0.91 ± 0.04	0.96 ± 0.03	0.94 ± 0.03	0.93 ± 0.03
	**4** **y**	**0.94** **±** **0.04**	**0.85** **±** 0.03*	**0.94** **±** **0.03**	**0.85** **±** 0.04*	0.89 ± 0.04	0.89 ± 0.04	0.87 ± 0.04	0.92 ± 0.04
HIRI	**0** **y**	1069 ± 42	1040 ± 41	1020 ± 41	1088 ± 41	1046 ± 41	1062 ± 41	**1027** **±** **41**	**1080** **±** **41**
	**4** **y**	958 ± 64	967 ± 61	947 ± 62	978 ± 62	986 ± 62	940 ± 62	**1012** **±** **62**	**914** **±** 62*
MISI	**0** **y**	**1.81** **±** **0.14**	**2.19** **±** **0.14**	2.15 ± 0.14	1.86 ± 0.15	2.31 ± 0.14	1.73 ± 0.14	1.99 ± 0.14	2.03 ± 0.14
(x10^2^)	**4** **y**	**2.02** **±** **0.15**	**1.79** **±** 0.14*	1.82 ± 0.15	1.98 ± 0.15	2.17 ± 0.15	1.65 ± 0.14	1.97 ± 0.15	1.83 ± 0.15
ISI	**0** **y**	**3.84** **±** **0.18**	**4.10** **±** **0.17**	3.99 ± 0.17	3.97 ± 0.17	4.13 ± 0.17	3.84 ± 0.17	3.96 ± 0.17	4.01 ± 0.17
	**4** **y**	**3.98** **±** **0.19**	**3.71** **±** 0.18*	3.83 ± 0.18	3.84 ± 0.18	4.20 ± 0.18	3.50 ± 0.18	3.86 ± 0.18	3.81 ± 0.18

Values are expressed as the mean ± SEM and were obtained by ANOVA for repeated measures. Low: Low circulating levels of miRNAs; High: High circulating levels of miRNAs. Statistical significance was evaluated by the Wilcoxon test using SPSS (now PASW Statistics for Windows (version 21.0) (IBM. Chicago, Illinois))

*Statistically significant differences (*p* < 0.05) are in bold

### Analysis of the probability of developing T2DM based on miRNAs levels using the Cox regression model

**Fig. 3:**
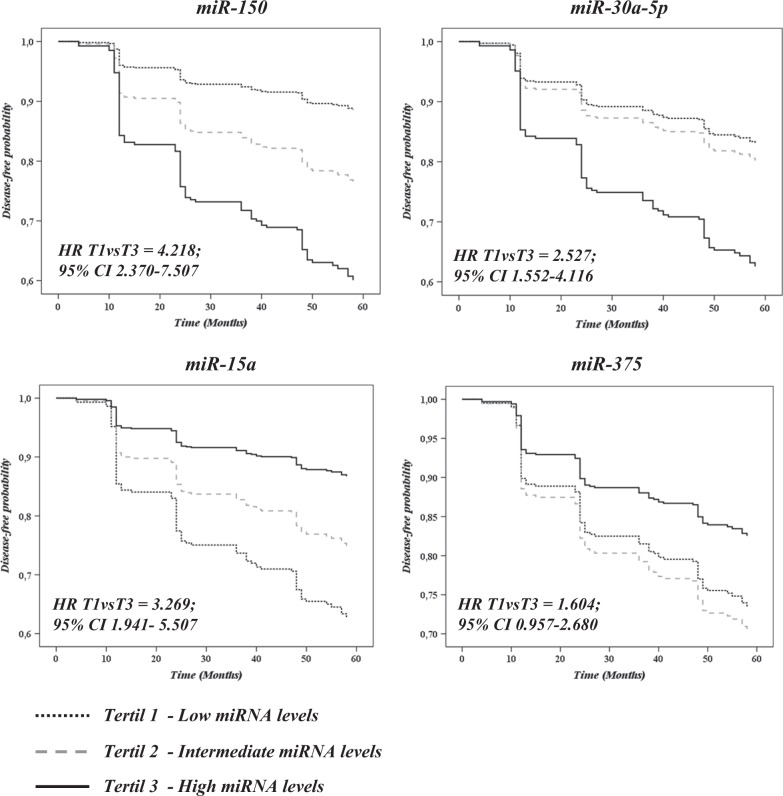
Disease-free survival by COX proportional hazards regression analysis of miRNAs. Subjects were divided into low (T1), intermediate (T2) and high (T3) baseline levels of miRNAs. The dotted black line represents subjects from T1, the dotted gray line represents subjects from T2, and the continuous black line represents subjects from T3. Analyses were adjusted for age, diet, gender, body mass index, glycosylated hemoglobin, HDL, triglycerides, and waist circumference

The risk of developing T2DM based on plasma miRNAs levels was evaluated by Cox regression analysis. We categorized patients by tertiles of the baseline levels for each miRNA: low levels (T1), intermediate levels (T2), and high levels (T3). Our results revealed that patients with high levels (T3) of *miR-150* and *miR-30a-5p* are at a higher risk of developing T2DM than those with low levels (T1) (*miR-150*, *HR* = 4.218; 95% CI: 2.370–7.507 and *miR-30a-5p*, *HR* = 2.527; 95% CI: 1.552–4.116). In contrast, patients with low levels (T1) of *miR-15a* and *miR-375* are at a higher risk of developing T2DM than those with high levels (T3) (*miR-15a*, *HR* = 3.269; 95% CI: 1.941–5.507 and *miR-375*, *HR* = 1.604; 95% CI: 0.957–2.687) (Fig. 3).

### Comparison between miRNAs-based model and established biomarkers of diabetic risk

**Fig. 4:**
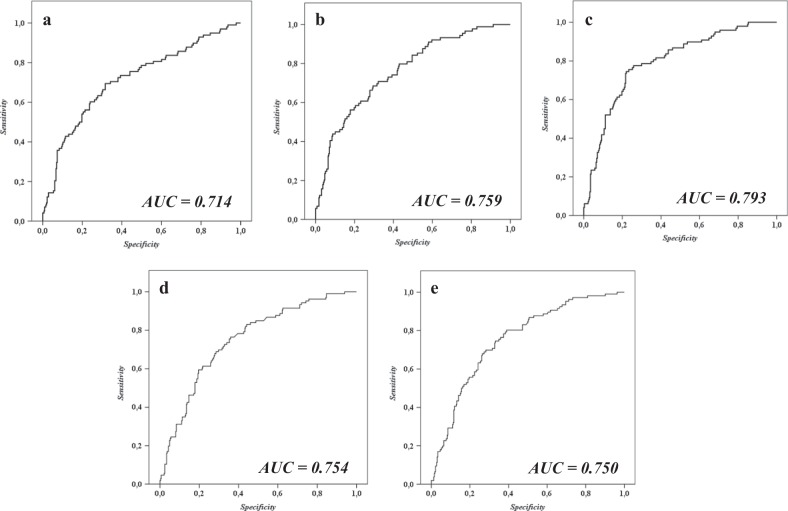
Receiver operating curves (ROC) analysis. **a** ROC model including clinical variables (age, gender, BMI, HDL-C, TG, HbA1c, fasting glucose and fasting insulin); (**b**) ROC model including clinical variables and OGTT-derived indexes (DI, MISI, ISI, HIRI, and IGI); (**c**) ROC model including clinical variables and studied miRNAs; (**d**) ROC model including studied miRNAs and FINDRISC score; and (**e**) ROC model including studied miRNAs and ADA score. The analyses were performed using SPSS (now PASW Statistics for Windows (version 21.0) (IBM. Chicago, Illinois))

We compared the predictive value of our model to the previously established biomarkers and FINDRISC and ADA scores in order to identify the added predictive power of the miRNAs. We performed a receiver operating characteristic (ROC) curve analysis, combining our model with the previously established biomarkers and scores. We observed an AUC of 0.714 when clinical variables were used (age, gender, BMI, HDL-C, TG, HbA1c, fasting glucose and fasting insulin), an AUC = 0.759 when we added the OGTT-derived index (DI, MISI, ISI, HIRI and IGI), an AUC = 0.793 when the clinical variables were combined with the miRNAs, an AUC of 0.754 when the FINDRISC score was combined with the miRNAs and an AUC of 0.750 when the ADA score was combined with the miRNAs (Fig. 4).

## Discussion

Our study showed that *miR-150* and *miR-30a-5p,* as well as *miR-15a* and *miR-375*, were deregulated in plasma several years before the diagnosis of T2DM. Specifically, we demonstrated for the first time that the baseline plasma levels of *miR-150* and *miR-30a-5p* were higher and *miR-15a* and *miR-375* were lower in incident-T2DM subjects compared with non-T2DM subjects after a median follow-up of 60 months, with intermediate levels in incident pre-DM and pre-DM subjects. Moreover, our study showed that a higher risk of developing T2DM is associated with low baseline plasma levels of *miR-15a* and *miR-375* and high baseline plasma levels of *miR-150* and *miR-30a-5p*. Our study also revealed the potential use of circulating miRNAs-based tools for predicting type 2 diabetes development in clinical practice, showing a small but higher added predictive value to the usual clinical variables used, such as age, gender, BMI, HDL-C, TG, HbA1c, fasting glucose and fasting insulin, than the predictive value added by indices derived from OGTT, such as DI, MISI, ISI, HIRI and IGI, to these variables.

Circulating miRNAs in human biofluids, such as blood^42^, have led to their use as non-invasive biomarkers for multiple pathologies, including CVD and T2DM^43–45^. This is based on the evidence that miRNAs play a major role in different mechanisms involved in T2DM development, such as insulin production, secretion and action, which supports their use as biomarkers for T2DM diagnosis^46^. However, the alteration in the miRNA pattern may precede or appear at early stages of diabetes or may be a consequence of the onset of diabetes^47^.

In this study, we have shown that the plasma levels of four miRNAs are deregulated in prediabetic states, several years preceding the development of T2DM, suggesting their potential use in the development of circulating miRNAs-based tools for T2DM prediction. When comparing the AUC of the ROC curves, we observed an improvement in the predictive capacity of the parameters used in clinical practice when the miRNAs were added to the model. However, this improvement was slightly higher than the improvement observed when OGTT-derived indexes were added to the clinical variables. Nevertheless, these findings suggest a potential use of miRNAs in clinical practice as they can be determined by a single blood collection, whereas OGTT requires continuous blood sampling (every half-hour for 2 h) to calculate the indexes.

In addition, the combined use of the FINDRISC and ADA scores with miRNAs yielded lower predictive power, probably because both scores include parameters not specific for T2DM and/or that they are self-reported, which diminishes their reliability and specificity for the prediction of T2DM status.

In fact, Cox proportional hazards regression analysis showed that patients with high levels of *miR-15a* and *miR-375* are at low risk of developing T2DM (HR 3.269 and 1.604, respectively) whereas patients with high levels of *miR-150* and *miR-30a-5p* are at high risk of developing T2DM (HR 4.218 and 2.527, respectively). The idea of the usefulness of miRNAs plasma levels to assess the risk of T2DM development is supported by other studies, in which alterations in plasma miRNAs levels have been observed before the development of T2DM^18,19^.

However, whereas the study by Zampetaki et al. is a case-control study in which matched controls were selected, our incidence study included all subjects from the CORDIOPREV cohort without T2DM at baseline, and we used all the non-T2DM patients after the follow-up period as controls.

Whereas our study evaluates the incidence of T2DM, the Zampetaki et al. study compares the differences between groups. The two study designs have their pros and cons: for example, in a case-control study, the recruitment of controls could be prone to selection bias—that is, the controls are systematically different from the population they are meant to represent. However, careful matching, aimed at eliminating any possible confounding factors, leads to efficiency in the study. Otherwise, incidence studies are usually the preferred approach for studying the causes of disease because they use all the available information on the source population over the risk period. However, they are often extremely costly in terms of time and resources, and equivalent results can be achieved more efficiently by using an incidence case–control study design. Although there is a difference in design, both studies demonstrate the potential of miRNAs as biomarkers of T2DM.

The study by Willeit et al. and the present work are incidence studies in which statistical and predictive analysis can be performed to evaluate the risk of T2DM development. Nevertheless, in contrast to the study by Willeit et al.^18^, which focused on *miR-122* to evaluate the risk of T2DM, our study included four miRNAs, which were combined with several clinical variables, such as age, gender, BMI, HDL-C, TG, HbA1c, fasting glucose and fasting insulin; this allowed us to build predictive models by adding miRNAs, which increased the predictive power of the traditional clinical parameters.

In addition, in our study, we followed ADA diagnostic criteria, whereas the studies by Zampetaki et al. and by Willeit et al. followed the World Health Organization guidelines, which do not take into account plasma levels of glycosylated hemoglobin as diagnostic criteria for diabetes, but it was used for the clinical validation of new cases of diabetes. Although these two methods are not very different, when the World Health Organization guidelines are followed, new cases of diabetes diagnosed by glycosylated hemoglobin are not detected.

Overall, *miR-150*, with a potential role in the main tissues for glucose homeostasis, and *miR-15a*, involved in insulin production and secretion, showed better risk assessment than *miR-30a-5p* and *miR-375*. However, analyses based on indexes of insulin signaling and release have shown that in patients with high circulating levels of *miR-150*, DI, ISI and MISI decreased during the follow-up period.

In fact, *miR-150* is highly expressed in the main tissues for glucose homeostasis, such as adipose, skeletal muscle and liver^13^. Moreover, this miRNA might come from hematopoietic cells, and the increase in *miR-150* could be a secondary phenomenon due to low-grade inflammation. This idea is supported by the involvement of this miRNA in the activation of B cells and other immune cells in adipose tissue, which, in turn, increases the insulin resistance of this tissue^37^. In addition, our study showed a relationship between *miR-150* plasma levels and OGTT-derived indexes, which are related to insulin signaling and release. However, further studies are needed to clarify which tissues are responsible for releasing *miR-150* into the blood stream and the mechanisms that regulate this miRNA.

In contrast, *miR-15a* positively regulates insulin biosynthesis by inhibiting endogenous UCP-2 (uncoupling protein-2) expression, leading to higher ATP levels in islets and improving glucose-stimulated insulin secretion (GSIS). This increase in intracellular ATP closes ATP-sensitive potassium channels, causing plasma membrane depolarization, the influx of Ca^2+^ and GSIS^35,48^. Thus, the lower levels of *miR-15a* that we observed in the patients who develop prediabetes and T2DM suggest that an impairment of stimuli for insulin secretion occurs before the development of T2DM. Moreover, our results are in line with previous studies, which have shown that circulating *miR-15a* levels decreased significantly before the onset of type 2 diabetes mellitus^14,19^.

Regarding miRNAs functions, the potential role of *miR-150* in insulin resistance in obesity by controlling adipose tissue inflammation seems to have a higher physiological impact than the change in insulin signaling by *miR-15a*, as suggested by the close relationship found between *miR-150* and the DI analyzed in our study. This idea is supported by the fact that the high plasma levels of *miR-30a-5p*, which have previously been shown to modulate beta cell function, were both associated with a decrease in DI. In fact, the overexpression of *miR-30a-5p* has been linked to the suppression of BETA2/NeuroD, which plays a key role in the regulation of insulin secretion and pancreatic beta cell dysfunction during glucotoxicity^12^. *Beta2/NeuroD* is a transcription factor that binds to the E element of the insulin gene^49^ and modulates K^+^ channels to regulate insulin secretion^50^.

Studies in animal models have shown the relationship of *miR-375* with the development of T2DM. In fact, *miR375KO* mice have a mass reduction in beta cells and an increase in the number of α cells, leading to islet instability^34^. Moreover, it has also been shown that the overexpression of *miR-375* in INS-E cells inhibits insulin gene expression in response to glucose by down-regulating the PDK1 gene, leading also to PI3K pathway inhibition^51^. In this sense, more research is needed to clarify the relationship between *miR-375* and the development of T2DM.

Certain limitations of the current study must be mentioned. The first limitation lies in the fact that we performed a focused bibliographic search of miRNAs associated with insulin sensitivity, insulin secretion and growth and proliferation of beta cells, and other potential T2DM-related miRNAs that have not been described were not included in our study. Another limitation lies in the fact that we analyzed plasma levels of miRNAs, and this approach, while suitable for assessing disease risk, may not accurately reflect the involvement and directionality in the interactions of these miRNAs in processes occurring at cellular levels. Moreover, the prevention of T2DM was not the primary endpoint of the CORDIOPREV trial but was a secondary analysis conducted in the subgroup of cardiovascular patients without T2DM at baseline (CORDIOPREV-DIAB study). The study included a large number of older patients with acute myocardial infarction, which limits our findings to people with these characteristics and precludes generalization to healthy people.

In conclusion, our study showed that deregulated plasma levels of *miR-150*, *miR-30a-5p*, *miR-15a*, and *miR-375* were observed years before the onset of T2DM and pre-DM and could be used to evaluate the risk of developing the disease, which may improve prediction and prevention among individuals at high risk of T2DM.
